# Differentiated Lithium Salt Design for Multilayered PEO Electrolyte Enables a High‐Voltage Solid‐State Lithium Metal Battery

**DOI:** 10.1002/advs.201901036

**Published:** 2019-09-19

**Authors:** Chen Wang, Tao Wang, Longlong Wang, Zhenglin Hu, Zili Cui, Jiedong Li, Shanmu Dong, Xinhong Zhou, Guanglei Cui

**Affiliations:** ^1^ Qingdao Industrial Energy Storage Research Institute Qingdao Institute of Bioenergy and Bioprocess Technology Chinese Academy of Sciences Qingdao 266101 P. R. China; ^2^ Center of Materials Science and Optoelectronics Engineering University of Chinese Academy of Sciences Beijing 100049 P. R. China; ^3^ College of Chemistry and Molecular Engineering Qingdao University of Science and Technology Qingdao 266042 P. R. China

**Keywords:** high voltage, Li metal, lithium salt, multilayered, poly(ethylene oxide) (PEO) electrolyte

## Abstract

Low ionic conductivity at room temperature and limited electrochemical window of poly(ethylene oxide) (PEO) are the bottlenecks restricting its further application in high‐energy density lithium metal battery. Herein, a differentiated salt designed multilayered PEO‐based solid polymer electrolyte (DSM‐SPE) is exploited to achieve excellent electrochemical performance toward both the high‐voltage LiCoO_2_ cathode and the lithium metal anode. The LiCoO_2_/Li metal battery with DSM‐SPE displays a capacity retention of 83.3% after 100 cycles at 60 °C with challenging voltage range of 2.5 to 4.3 V, which is the best cycling performance for high‐voltage (≥4.3 V) LiCoO_2_/Li metal battery with PEO‐based electrolytes up to now. Moreover, the Li/Li symmetrical cells present stable and low polarization plating/stripping behavior (less than 80 mV over 600 h) at current density of 0.25 mA cm^−2^ (0.25 mAh cm^−2^). Even under a high‐area capacity of 2 mAh cm^−2^, the profiles still maintain stable. The pouch cell with DSM‐SPE exhibits no volume expansion, voltage decline, ignition or explosion after being impaled and cut at a fully charged state, proving the excellent safety characteristic of the DSM‐SPE‐based lithium metal battery.

## Graphical abstract

**A multilayered poly(ethylene oxide)‐based electrolyte** is exploited to achieve excellent cycling performance for a high‐voltage LiCoO_2_ Li metal battery. A delicately designed salt combination and proportion is presented as the multilayer, boosting wide electrochemical working window, high ionic conductivity, and suppression of Li dendrites simultaneously.

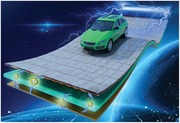

## 1 Introduction

High‐energy density lithium batteries have been studied extensively in the past four decades, due to the increasingly demand of advanced electronic devices, electric vehicles, and grid‐scale storage.^1^ Li metal is intensively regarded as the most promising anode material for high‐energy batteries because of its lowest potential (−3.040 V vs standard hydrogen electrode) and high specific energies (3860 mAh g^−1^).^2^, ^3^, ^4^, ^5^ Unfortunately, the dynamic Li plating/stripping on Li metal anode suffers from side reactions between liquid electrolyte and Li metal, inducing the formation of heterogeneous, unstable solid electrolyte interphase (SEI) layer, and the growth of Li dendrites.^6^, ^7^, ^8^ Li dendrites and resultant “dead” Li give rise to severe safety hazards, including short circuit, thermal runaway, and combustion/explosion.^9^, ^10^, ^11^ In this respect, it is imperative to find a way to suppress the growth of Li dendrite and promote the safety characteristic. In recent years, solid polymer electrolyte (SPE) has been widely investigated as one of the most promising solutions, which exhibits several advantages such as excellent mechanical flexibility, easier fabrication, reinforced adhesion to electrode.^12^, ^13^, ^14^, ^15^, ^16^ Compared with inorganic solid electrolyte, SPE has made a successful commercial demonstration in solid‐state lithium batteries, as its manufacturing process fits well with the present commercialized Li‐ion battery industries.^17^, ^18^, ^19^, ^20^, ^21^, ^22^

Poly(ethylene oxide) (PEO)‐based electrolyte has been the most intensively investigated candidate due to its low glass transition temperature (*T*_g_) and outstanding performance for “solvating” alkaline salts.^23^ Wright and co‐workers discovered that PEO could be used as an ionic conductor when combined with alkaline salt in 1973.^24^ The first commercial demonstration of solid‐state lithium battery was also employed PEO as the matrix of electrolyte.^25^ However, the low ionic conductivity and poor high‐voltage stability severely hindered their further commercialization process.^26^, ^27^, ^28^, ^29^, ^30^ Among tremendous efforts, plasticizing is one of the effective strategies by far to increase the ionic conductivity up to 10^−3^ S cm^−1^ level.^31^, ^32^, ^33^, ^34^ The combination of PEO and succinonitrile (N≡C—CH_2_—CH_2_—C≡N, abbreviated as SN), a typical plastic crystal, has been reported to deliver a high ionic conductivity of 2.9 × 10^−3^ S cm^−1^ at ambient temperature.^35^ However, this composite matrix of PEO and SN has been always precluded as SPE for high‐voltage battery. Although SN possessed high‐voltage tolerance, the poor stability of PEO under high‐voltage utilization remained. Furthermore, a parasitic reaction between SN and Li metal was demonstrated to keep on occurring at the interface, and the byproducts from this reaction could not form a stable dense solid‐state interphase on lithium metal anode.^36^, ^37^, ^38^ Hence, a PEO‐based SPE with wide potential window is still urgently required.

Surface modification and multilayer building are two primary and efficient methods of improving the antioxidant stability of systems with PEO‐based electrolytes.^39^, ^40^, ^41^, ^42^ It is reported that LiCoO_2_ coated with Li_1.4_Al_0.4_Ti_1.6_(PO_4_)_3_/Li metal battery with PEO‐based electrolyte exhibits 93% discharge capacity retention after 50 cycles at the charging cut‐off voltage of 4.2 V.^41^ Zhou et al. have constructed a double‐layer PEO‐based polymer electrolyte, the LCO/Li metal battery has a capacity of 91.2% of the highest discharge capacity after 100 cycles at 4.25 V with the temperature of 65 °C.^42^ The cycling performance of high‐voltage LiCoO_2_/Li batteries with PEO‐based SPE so far is shown as Table S1 of the Supporting Information. Herein two mechanisms can be proposed to broaden the potential window of certain electrolyte according to the above two methods. One is tuning the frontier orbital energy level of electrolyte thermodynamically. The interaction between polymer segments and salts (both cations and anions) can modify the HOMO and/or LUMO of electrolyte. The other one is building a kinetically stable interphase at cathode surface to restrain the direct contact of electrolyte with charged cathode materials. The anions can migrate to the electrode and contribute to the formation of a stable cathode electrolyte interphase (CEI)/SEI. Therefore, it is rational to say that salt designing and multilayer building are crucial to construct a stable PEO‐based SPE toward both high‐voltage cathode and highly reactive lithium metal anode.

In this work, we introduced a lithium salt with bulk anion which was first synthesized by us, namely, lithium trifluoro (perfluoro‐tert‐butyloxyl) borate (Li[(CF_3_)_3_COBF_3_], LiTFPFB),^43^ and construct a PEO‐based multilayered solid polymer electrolyte (DSM‐SPE). This lithium boron salt was employed in the cathode and anode contacting layer, contributed to a stable SEI/CEI at both cathode and lithium anode interfaces. In the meantime, the intermolecular interaction between two kind salts and PEO segments contributed to a lower HOMO of SPE. Compared to general PEO‐based SPE, this DSM‐SPE presented excellent cycling stability of a 4.3 V‐class LiCoO_2_/Li metal batteries with a capacity of 125 mAh g^−1^ after 100 cycles with current density of 0.1 C at 60 °C (2.5–4.3 V), corresponding to 83.3% of the initial capacity (150 mAh g^−1^), which is the best cycling performance of high‐voltage LiCoO_2_/Li batteries with PEO‐based SPE so far.^44^ Moreover, the Li/Li symmetrical cells exhibited stable and low polarization plating/stripping curves (less than 80 mV over 600 h) at current density of 0.25 mA cm^−2^ and 0.25 mAh cm^−2^. Even under high current density utilization of 2 mA cm^−2^ with capacity of 2 mAh cm^−2^, a stable and smooth profile could still be obtained.

## 2 Results and Discussion

### 2.1 Construction of the DSM‐SPE

**Figure 1:**
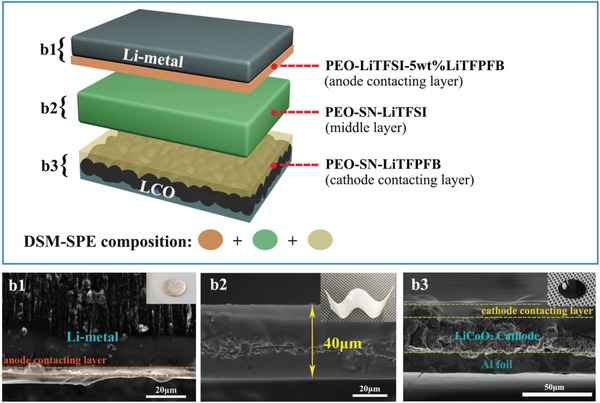
Schematic of differentiated salt‐based multilayered solid polymer electrolyte (DSM‐SPE). The bilateral layers are in situ formed on the surfaces of electrodes. The interface SEM images of section view of b1) Li metal anode with anode contacting layer, b2) middle layer SPE, and b3) LiCoO_2_ cathode with cathode contacting layer; inset shows the digital photos of them, respectively.

The schematic of DSM‐SPE is shown in **Figure** **1**. To achieve the high ionic conductivity of the electrolyte, primary middle layer SPE is designed with single lithium salt of lithium bis(trifluoromethanesulfonyl)imide (LiTFSI) (PEO, SN, LiTFSI with mass ratio of 7:7:6). TFSI^−^ ions in LiTFSI are composed of N atom with high electronegativity and two S atoms connected to CF_3_ with high electron‐withdrawing capacity. This structure is contributed to high dissociation by dispersing negative charge, delivering high ionic conductivity of SPE with LiTFSI.^27^ Then, in order to construct a wide electrochemical window standing polymer electrolyte, the LiTFPFB is targeted used in bilateral layers. Cathode contacting layer is composed of PEO, SN, and LiTFPFB (with mass ratio of 7:7:6). Anode contacting layer is composed of PEO, LiTFSI (with mass ratio of 3:1), and plus 5 wt% LiTFPFB. The employment of LiTFPFB can improve the stability and compatibility with cathode, protect Al current collector from corrosion at high voltage up to 4.5 V at cathode side, and assist in the formation of stable solid electrolyte interphase (SEI), prevent the side reaction between Li metal/SN at anode side.^43^, ^45^

In a typical process, the middle layer SPE membrane is fabricated by the solution‐casting method (explained at the Experimental Section). From the cross‐section scanning electron microscope (SEM) image of this middle layer (Figure 1(b2)), we can see the thickness is ≈40 µm. It is proved that the integrity, bendability, and flexibility of it (Figure 1(b2); Figure S1, Supporting Information) with a high compact structure and smooth surface (Figure S2, Supporting Information). The structural difference of this middle layer (PEO‐SN‐LiTFSI) with PEO‐LiTFSI was detected by X‐ray diffraction (XRD), as shown in Figure S3 of the Supporting Information. There are several intense characteristic peaks in the XRD spectra of PEO‐LiTFSI electrolyte, indicating the high crystallinity of PEO at room temperature. However, there is only a broad peak in PEO‐SN‐LiTFSI, proving the addition of SN reduces the crystallinity. This amorphous structure facilitates the fast mobility of the polymer chains, promoting the migration of Li^+^ ion to a big extent.

The bilateral layers are in situ formed by directly casting polymer on electrodes (explained at the Experimental Section). This in situ method can commendably reinforce the interfacial adhesion between electrolyte and cathode. Typical SEM images of interfaces between Li metal/anode contacting layer and LiCoO_2_/cathode contacting layer are shown in Figure 1(b1),(b3), respectively. From the digital photographs of electrodes with coatings shown in the insets of Figure 1(b1–b3), we can see the bilateral layers of DSM‐SPE present excellent tight contact to electrodes with thickness of 9.5 µm (cathode contacting layer) and 5 µm (anode contacting layer). Explicitly, the thickness of DSM‐SPE is about 55 µm. The bilateral layers are all controlled less than 10 µm, in case of severe reduction of ionic conductivity.

### 2.2 Physical and Electrochemical Characterizations of DSM‐SPE

**Figure 2:**
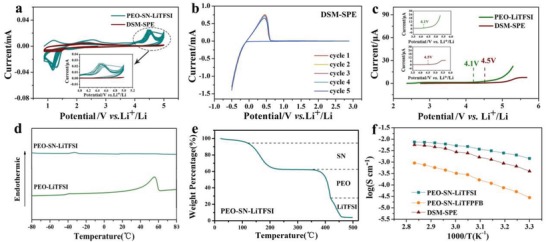
a) CV plots of the Li metal/stainless‐steel batteries of PEO‐SN‐LiTFSI and the DSM‐SPE at a scan rate of 0.5 mV s^−1^, using stainless steel as the working electrode, Li as the counter and reference electrode. b) Stability and reversibility of Li stripping and plating of DSM‐SPE. c) LSV of PEO‐LiTFSI and the DSM‐SPE, inset shows the magnification part. d) DSC profiles of SN, PEO‐LiTFSI, and PEO‐SN‐LiTFSI. e) TGA thermogram of PEO‐SN‐LiTFSI. f) The temperature‐dependent ionic conductivity of PEO‐SN‐LiTFSI, PEO‐SN‐LiTFPFB, and DSM‐SPE.

The electrochemical stability of SPE is of vital importance for potential practical application. The antioxidation property and stability of the SPEs were evaluated by cyclic voltammetry (CV) test in a stainless steel (SS)/Li metal cell at a scan rate of 0.5 mV s^−1^ from 0.8 to 5 V, as shown in **Figure** **2**a. The onset potential of DSM‐SPE for oxidative current is higher compared with PEO‐SN‐LiTFSI. This result indicated that the DSM‐SPE could effectively broaden the potential window of SPE. In addition, the lithium deposition and stripping stability at lower voltage was scanned with PEO‐SN‐LiTFSI and DSM‐SPE from open‐circuit voltage to −0.5 V, which are shown in Figure S4 (Supporting Information) and Figure 2b. The currents of repeated cycles in Figure 2b are fairly constant with that of the initial peak of CV curves, indicating that the plating/stripping of lithium are highly reversible in DSM‐SPE system. While the CV curves of lithium deposition and stripping scanned with PEO‐SN‐LiTFSI fluctuated drastically due to the continuous side reaction between SN and Li metal. By a linear sweep voltammetry (LSV) scan of the PEO‐LiTFSI and DSM‐SPE that sandwiched between SS and Li metal plates (Figure 2c), we can see the electrochemical window of DSM‐SPE is wider than that of PEO‐LiTFSI. A significant anodic current increase is observed in PEO‐LiTFSI‐based cell at 4.1 V, while the DSM‐SPE exhibits no observable decomposition behavior until 4.5 V in a fair comparison.

Differential scanning calorimetry (DSC) measurements are employed to investigate the amorphousness behavior of the SPE, shown in Figure 2d. PEO shows reduced Tm of 57 °C after the addition of LiTFSI. With further introduction of SN, PEO‐SN‐LiTFSI showed no characteristic peak of PEO or SN, proved the completely amorphous state of it. Thermal safety is of vital importance for energy storage materials. Herein the thermogravimetric analysis (TGA) thermogram of PEO‐SN‐LiTFSI was tested under a flow of N_2_ at a rate of 10 °C min^−1^ (Figure 2e). PEO‐SN‐LiTFSI SPE did not exhibit an obvious weight loss until 120 °C, indicating that the PEO‐SN‐based electrolyte film could be utilized at the elevated temperature up to 120 °C without significant deterioration. In addition, the flame tests of glass fiber‐based liquid electrolyte and cellulose‐based PEO‐SN‐LiTFSI SPE are implemented to further evaluate the safety property of the SPE, shown as Figure S5 of the Supporting Information. It can be seen that glass fiber soaked with liquid electrolyte can be easily ignited until it burned out, while the flame of cellulose‐based PEO‐SN‐LiTFSI electrolyte extinguished right after being deviated from fire.

To demonstrate the advantage of particular lithium salt design in DSM‐SPE, we fabricated PEO‐SN‐LiTFPFB SPE in which LiTFPFB totally replaced LiTFSI and measured the temperature‐dependent ionic conductivity for comparison. Figure 2f presents the Arrhenius plots for the conductivity of PEO‐SN‐LiTFSI, PEO‐SN‐LiTFPFB, and DSM‐SPE. For the middle layer SPE with single salt of LiTFSI (PEO‐SN‐LiTFSI), high ionic conductivity of 1.1 × 10^−3^ S cm^−1^ at 30 °C was observed. As to the single LiTFPFB‐based SPE, ionic conductivity decreased by more than one order, especially at room temperature (0.28 × 10^−4^ S cm^−1^). The temperature‐dependent conductivity of DSM‐SPE (0.5 × 10^−3^ S cm^−1^ at 30 °C) also decreased compared to PEO‐SN‐LiTFSI, while it is much higher than that of PEO‐SN‐LiTFPFB. The purpose of designing differentiated salt in different layers rather than totally replace LiTFSI with LiTFPFB is to get higher ionic conductivity in the guarantee of interfacial stability.

### 2.3 Performance and Characterization of Li/Li Symmetrical Cells

**Figure 3:**
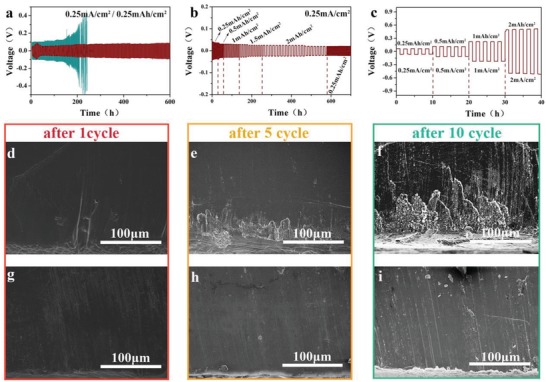
a) Lithium striping and plating curves of the Li/Li symmetrical cells with middle layer SPE(blue) and anode contacting layer/middle layer/anode contacting layer SPE(red) at current density of 0.25 mA cm^−2^ (60 °C). b) Lithium striping and plating curves of the Li/Li symmetrical cells with anode contacting layer/middle layer/anode contacting layer SPE at current density of 0.25 mA cm^−2^ with areal capacity from 0.25 to 2 mAh cm^−2^ (60 °C). c) Rate performance of the Li/Li symmetrical cells measured under different current density with anode contacting layer/middle layer/anode contacting layer SPE; The cross‐sectional SEM images of Li metal disassembled from the Li/Li symmetrical cells d–f) with middle layer SPE and g–i) with anode contacting layer/middle layer/anode contacting layer SPE after 1, 5, and 10 cycles, respectively.

**Figure 4:**
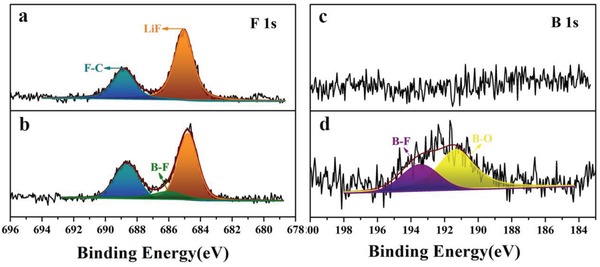
XPS characterization of the SEI film formed on cycled Li anodes after 50 cycles with a,c) middle layer SPE and b,d) anode contacting layer/middle layer/anode contacting layer SPE.

To avoid the direct contact between SN and Li metal anode, Li/Li symmetric cells were assembled with anode contacting layer/middle layer/anode contacting layer sandwich electrolyte. Lithium‐ion transference number (tLi^+^) is representative of the transportation of lithium ions, which is highly desirable in lithium‐ion battery applications. Herein the tLi^+^ of the SPE is calculated to be 0.56, shown in Figure S6 of the Supporting Information, which was higher than that of conventional liquid electrolytes. The entrapment of the large bis(trifluoromethane) sulfonamide anions within the polymer network matrices could be account for the high tLi^+^ of the SPE. The lithium stripping and plating performance was measured by the polarization of Li/Li symmetrical cells at 0.25 mAh cm^−2^ with the current density of 0.25 mA cm^−2^ at 60 °C. As shown in **Figure** **3**a, the overpotential of deposition or dissolution of Li in Li/Li symmetrical cell with middle layer SPE is 100 mV at the beginning and increases drastically with time increasing. Eventually, this cell exhibits a large and irreversible voltage drop at 250 h, which is induced by the parasitic reaction between SN/Li metal anode and the growth of dendrite. Lithium stripping and plating curve of single salt Li/Li symmetrical cell with PEO‐LiTFSI/PEO‐SN‐LiTFSI/PEO‐LiTFSI is shown in Figure S7 of the Supporting Information. The overpotential of deposition or dissolution of Li in this symmetrical cell exhibits short circuit at 220 h after persistent instability. On the other hand, the Li/Li symmetrical cell with anode contacting layer/middle layer/anode contacting layer SPE(PEO‐LiTFSI‐5 wt% LiTFPFB/PEO‐SN‐LiTFSI/PEO‐LiTFSI‐5 wt% LiTFPFB) exhibits a low polarization voltage (less than 80 mV) and a long cycling life (over 600 h) after a slight increase of overpotential at the beginning. It is speculated that there is SEI formed at the initial stage, inducing the increasing of overpotential at the beginning and stabilizing of the polarization soon afterward. Figure 3b is the cycling stability of Li/Li symmetrical cell with anode contacting layer/middle layer/anode contacting layer SPE at current density of 0.25 mA cm^−2^ with capacity changing. The overpotential of charge/discharge is stable despite capacity changes up to 2 mAh cm^−2^. The first 50 h of the lithium plating and stripping curves of Figure 3a,b were zoomed in and shown in Figure S8 of the Supporting Information. Rate capability of Li/Li symmetrical cell with anode contacting layer/middle layer/anode contacting layer SPE is also shown as **Figure** **4**c, the polarization curve is steady in each 5 cycles test from low capacity of 0.25 mAh cm^−2^ to high capacity of 2 mAh cm^−2^. To obtain the interface distinction between Li/middle layer SPE and Li/ anode contacting layer SPE, the SEM measurement was used to scrutinize the morphology of Li metal after 1, 5, and 10 cycles. Cross‐section SEM images of Li metal disassembled from cells with middle layer SPE and anode contacting layer/middle layer/anode contacting layer SPE were shown as Figure 4d–i, with noticeable differences, the enlargement images were shown in Figure S9 of theSupporting Information. For the cell with middle layer SPE, there were massive byproducts generated along with cycling. On the other hand, the cross‐section of Li metal electrode cycled with anode contacting layer/middle layer/anode contacting layer SPE presented uniform morphology without lumps and cracking, suggesting that the anode contacting layer contact to Li metal electrode prevents Li metal from corrosion effectively. The SEM images of the surfaces of Li metal after 1, 5, and 10 cycles are shown as Figure S10 of the Supporting Information. Unlike the uneven nubbly products formed on the surfaces of Li metal directly contact to middle layer SPE, there are smoothand uniform surfaces of Li metal with anode contacting layer. Meanwhile, to visually prove the protection of 5 wt% LiTFPFB for Li metal, the interfacial stability experiments were carried out. Li metal was directly contact to PEO‐SN, PEO‐SN‐LiTFSI (middle layer SPE), and PEO‐LiTFSI‐5 wt% LiTFPFB (anode contacting layer SPE), shown as Figure S11 of the Supporting Information. After 24 h, there are obvious yellow products produced at the interfaces of the first two samples, due to the side reactions between SN and Li metal. While there is no color change of the third Li metal, suggesting the formation of a stable passivation layer and suppression of side reactions between SN and Li metal by anode contacting layer SPE.

X‐ray photoelectron spectroscopy (XPS) is further employed to investigate the components of the SEI film. After disassembling of the cells after 50 cycles, Li metal was immersed in tetrahydrofuran (THF) for 3 h to dissolve the residual SPE adhered to it prior to the XPS measurement. F 1s, B 1s, C 1s, and O 1s XPS spectra of Li metal contact to middle layer SPE and anode contacting layer/middle layer/anode contacting layer SPE are shown as Figure 4 and Figure S12 (Supporting Information). In the process of soaking, the organic ingredients may be dissolved by THF, while the inorganic ingredients we analyzed of SEI film would not be changed. Hence the surface chemistry of two groups is compared to each other with standard of inorganic constituent. The binding energies are calibrated with C1s at 284.8 eV. A considerable amount of LiF (684.5 eV, F1s) are detected on both surfaces of this two kind Li metal anodes,^46^, ^47^ which is belonging to the decomposition of lithium salts.^48^ In addition, B–F (686.6 eV, F1s; 193.6 eV, B 1s) and B–O (191.3 eV, B 1s) are observed in the anode contacting layer/middle layer/anode contacting layer SPE‐based cell besides Li–F, which originate from the —CF_3_ and —BF_3_ groups of TFPFB^−^ anion.^49^ XPS spectra therefore suggest the complex reactions at the interface between Li metal and SPE, proving the participation of fluoroalkoxyl groups in the formation process of SEI, which can significantly enhance the interface stability of Li metal anode.

### 2.4 Performance and Characterization of LiCoO_2_/Li Metal Batteries

**Figure 5:**
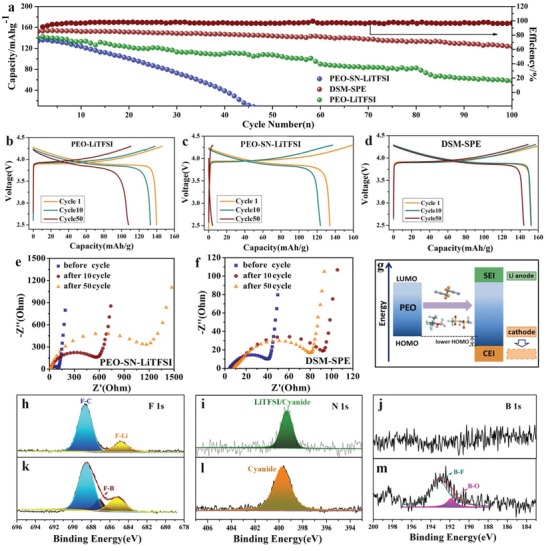
a) The cycling performance of LiCoO_2_/Li batteries with middle layer SPE, DSM‐SPE, and PEO‐LiTFSI. The charge/discharge curves of the 1st cycle, 10th cycle, and 50th cycle of LiCoO_2_/Li batteries with b) PEO‐LiTFSI, c) middle layer SPE(PEO‐SN‐LiTFSI), and d) DSM‐SPE. The resistance of LiCoO_2_/Li batteries with e) PEO‐SN‐LiTFSI and f) DSM‐SPE before and after 10 cycles, 50 cycles. g) Scheme representative of the interfacial stability of cathode enabled by the lower HOMO of DSM‐SPE and CEI. Surface chemistries of the LiCoO_2_ cathodes with h–j) middle layer SPE and k–m) DSM‐SPE after 10 cycles.

Significantly improved cycling performance was obtained in the LiCoO_2_/Li metal batteries with DSM‐SPE. The galvanostatic discharge curves of LiCoO_2_/Li batteries with middle layer SPE (PEO‐SN‐LiTFSI) , DSM‐SPE, and bare PEO‐based SPE (PEO‐LiTFSI) at a current density of 0.1 C (1 C = 155 mAh g^−1^) with voltage range of 2.5–4.3 V at 60 °C are shown as **Figure** **5**a. The cycling test of the LiCoO_2_/Li battery with the middle layer SPE shows a continuous capacity fading even more drastically than with PEO‐LiTFSI during the first 10 cycles before the fluctuation and quick decay (Figure 5a), which may due to increasing resistance of interface caused by side reactions between SN and Li metal. Nevertheless, the discharge capacity of the LiCoO_2_/Li battery with DSM‐SPE is still 127 mAh g^−1^ after 100 cycles, corresponding to 83.5% of the initial capacity (152 mAh g^−1^). The discharge capacity retention of the DSM‐SPE is 95% after 50 cycles, absolutely beyond other kind of batteries (Figure 5b–d). It is worth noting that the coulombic efficiency rapidly increased to reach a constant value of about 97% after the initial 5 cycles and remained stable throughout the cycling, indicating high ionic plating/stripping efficiency of this DSM‐SPE during cycling. The discharge capacity of LiCoO_2_/Li battery with DSM‐SPE at 0.1 C, 0.2 C, 0.5 C is 150, 146, and 119 mAh g^−1^, respectively, and presents a decent rate capability (Figure S13, Supporting Information). The resistance of LiCoO_2_/Li batteries before cycle and after 10 cycles, 50 cycles with PEO‐SN‐LiTFSI and DSM‐SPE are shown in Figure 5e,f, respectively. The resistance of LiCoO_2_/Li battery with DSM‐SPE before and after cycling is much smaller than that with PEO‐SN‐LiTFSI SPE, indicating the reinforced contact between cathode and DSM‐SPE. It is worth noting that the resistance of DSM‐SPE‐based battery after 50 cycles decreased a little compared to that after 10 cycles due to the formation of protective CEI film. Hence we can get that the SEI layer and CEI layer generated in the DSM‐SPE‐based battery is more stable and less resistive than that generated with PEO‐SN‐LiTFSI along with cycling.

Herein the high‐voltage kinetic and thermodynamic stability of DSM‐SPE were respectively investigated. The surface chemistry of the LiCoO_2_ cathodes with PEO‐SN‐LiTFSI and DSM‐SPE after 10 cycles is measured by XPS (shown as Figure 5h–m; Figure S14, Supporting Information). Two peaks at 286.8 eV (C—O—C) and 284.8 eV (C—C/C—H) are observed at both C 1s curves. Different from the spectra of cathode directly contact to middle layer SPE, pronounced peaks for B–O (191.7 eV), B–F (193 eV, B1s; 687.2 eV, F1s), F–Li (684.8 eV, F 1s) are all belong to the decomposition products of LiTFPFB in DSM‐SPE. Peak of cyanide (399.5 eV, N 1s) comes from SN of the electrolyte. The strong interaction between SN and borate groups, synergistically contribute to the formation of uniform coverage of cathode electrolyte interface (CEI) layer,^50^ improving the interfacial stability.

Additionally, Fourier transform infrared spectroscopy (FTIR) spectra of PEO‐SN, PEO‐SN‐LiTFPFB (cathode contacting layer), and PEO‐SN‐LiTFSI(middle layer) are respectively measured to further investigate the intermolecular interactions between PEO, SN with different lithium salts (shown as Figure S15, Supporting Information). The peaks at 2891 cm^−1^ assigned to C—H stretching of PEO all became broader and a shoulder appeared in higher wavenumber side after the addition of lithium salts. The characteristic peak of C—O—C displayed a red shift from 1110 to 1097 cm^−1^ after the addition of LiTFSI, and shifted to a lower wavenumber at 1084 cm^−1^ after the lithium salt is changed to LiTFPFB. Particularly the C—O—C of PEO becomes broader and nearly combines into their corresponding peaks. These results confirm the complexation between the ether oxygen of PEO and cation(Li^+^), and which is stronger in PEO‐SN‐LiTFPFB.^51^ On the basis of complexation with Li^+^ of lithium salts, the ether oxygen donates the lone pairs to Li^+^, resulting in the lowering of the HOMO energy level of PEO.^52^ The scheme representative of the interfacial stability of cathode enabled by the lower HOMO of DSM‐SPE and CEI is shown in Figure 5g.

Combining the XPS and FTIR spectra with cycling performance, we can conclude that the synergistic effect of functional lithium borate salt and SN contribute to the formation of uniform coverage of cathode electrolyte interface (CEI) layer; the interactions between PEO and Li^+^ could broaden the HOMO–LUMO gap of the electrolyte. That is to say, the particular lithium salt design dedicates to the excellent cycling stability of LiCoO_2_/Li metal battery at high voltage by improving both kinetic compatibility and thermodynamic stability.

### 2.5 Safety Evaluation of LiCoO_2_/Li Pouch Cell with DSM‐SPE

**Figure 6:**
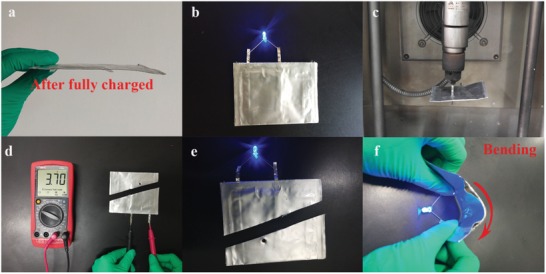
Safety test of the LiCoO_2_/Li pouch cell with DSM‐SPE. a) Digital photo of the pouch cell with DSM‐SPE after fully charged. b) The working cell to light up an LED device. c) Nail test for the pouch cell. d) Voltage detection of the pouch cell after nail test and corner cut test. e,f) Pouch cell can still lighten the LED after nail test and corner‐cut test at normal and bending states.

To demonstrate the high safety and potential practical application of this DSM‐SPE, pouch cell was assembled with LiCoO_2_ cathode of industrial level loading of 34.5 mg cm^−2^, and fully charged to 4.3 V. Same as coin cells with DSM‐SPE, the cathode contacting layer and anode contacting layer are in situ formed on surfaces of LiCoO_2_ cathode and Li metal anode beforehand, too. Digital photos of the all‐solid state internal structure and external appearance after packaging of the pouch cell with DSM‐SPE are shown as Figure S16 of the Supporting Information. After fully charged, there was no volume expansion, ignition or explosion detected for the cell, shown as **Figure** **6**a. The pouch cell can light up a blue LED lamp under a normal condition. After that, nail test (Figure 6c) and corner‐cut (Figure 6d) test are applied to further evaluate the safety performance of the DSM‐SPE and the cell exhibit almost no decline in voltage after these two processes. It is worth mentioning that the cell after cut can still lighten the blue LED at normal and bending state, shown as Figure 6e,f, respectively. In order to further elucidate the security property, the events during heating process has been evaluated by the accelerating rate calorimeter (ARC). The reactor of the main component of the ARC test stand is shown in Figure S17 of the Supporting Information. Three LCO/Li metal pouch cells of 3Ah with PEO‐LiTFSI SPE, DSM‐SPE, and liquid electrolyte (1 m EC/DMC‐LiPF_6_) have been assembled, charged to 4.3 V and measured by ARC. The temperature versus time plots of the LCO/Li metal pouch cells with three kind electrolytes are shown as Figure S18 of the Supporting Information. First, it can be seen that the pouch cell with liquid electrolyte exhibited thermal runaway after continuous internal exothermic reaction, while both pouch cells with PEO‐LiTFSI and DSM‐SPE did not exhibit thermal runaway. The pouch cell with liquid electrolyte exploded eventually due to the yield of gas or short circuit, while the pouch cells of first two groups did not exhibit any volume expansion. Second, internal exothermic reactions are all detected from about 60 °C in the pouch cells with PEO‐LiTFSI and DSM‐SPE. While the exothermic process (highlighted with blue) stops automatically after a period of time. We speculate that the exothermic processes are happened at the interfaces, because there is no stable CEI or SEI film formed only after the first cycle of charging to 4.3 V. To sum up, the addition of SN in DSM‐SPE does no harm to the security property of the system, and the DSM‐SPE‐based system is much safer than that with liquid electrolytes.

## 3 Conclusions

Herein, we constructed a differentiated lithium salt‐based multilayered SPE. This DSM‐SPE is composed of three layers and has taken advantages of every layer. Primary middle layer promises high ionic conductivity of the whole electrolyte, bilateral layers remarkably improve the interfacial contact and accelerate the formation of stable SEI and CEI films. Compared with traditional PEO‐LiTFSI and middle layer SPE, this DSM‐SPE presented extremely ameliorative performance in high‐voltage LiCoO_2_/Li battery and stripping/plating of lithium. The LiCoO_2_/Li battery with DSM‐SPE can deliver discharge capacities of 150, 146, and 119 mAh g^−1^ at 0.1 C, 0.2 C, 0.5 C from 2.5 to 4.3 V, respectively, at 60 °C. A stable cycling over 100 cycles with 83.3% capacity residual was detected at 0.1 C. Moreover, the Li/Li symmetrical cells presented a low voltage polarization as well as a smooth and dendrite free surface at a current density of 0.25 mAh cm^−2^ for more than 600 h and maintained steady cycling at areal capacity up to 2 mAh cm^−2^. This facile design philosophy could be used to solve interfacial problems between electrolyte and electrode, widen the electrochemical window and extensively applied to other solid‐state Li metal batteries.

## 4 Experimental Section

*Materials*: PEO (Mw = 300 000) used in this work was purchased from Alfa Aesar. LiTFSI, lithium difluoro(oxalato)‐borate (LiTFPFB), and SN (all in battery‐grade purity) were all obtained from Sigma Corporation.

*Fabrication of the DSM‐SPE*: The middle layer SPE was fabricated by a facile slurry casting–drying method. The homogeneous solution was prepared by dissolving PEO, SN, and LiTFSI with the mass of 0.7, 0.7, and 0.6 g into 8 g anhydrous THF and then casted on a cellulose nonwoven. After the evaporation of the solvent in a vacuum oven at 60 °C for 12 h, the electrolytes were peeled off and punched into disks with a diameter of 16.5 mm and further dried at 60 °C under vacuum for 12 h. The thickness of the resulted cellulose‐supported middle layer SPE is 40 µm.

The electrode contacting layers were fabricated by in situ formation method in glove box. First, solution was prepared by adding PEO, SN, and LiTFPFB with the mass of 0.35, 0.35, and 0.3 g into 7 g THF and stirred at 60 °C until complete dissolution. Then 50 µL precursor solution was dripped onto LiCoO_2_ cathode and the cathode contacting layer was in situ formed on the surface of LiCoO_2_ cathode. Similarly, PEO, LiTFSI, and 5 wt% LiTFPFB with mass of 0.3, 0.1, and 0.02 g were added into 7 g THF. After complete dissolution, 40 µL precursor solution was dripped onto Li metal anode and the anode contacting layer was in situ formed on the surface of Li metal anode. Finally, the cathode and anode with contacting layers were placed in the argon‐filled glove box for 24 h for the totally volatilization of THF.

*Preparation of Solid Lithium Metal Battery with DSM‐SPE*: The laminate of LiCoO_2_ electrode was prepared by casting a slurry mixture containing 80 wt% active material, 10 wt% super P, 10 wt% polyvinylidene fuoride binder in *N*‐methyl‐2‐pyrrolidone onto an aluminum (Al) current collector foil. After drying, the electrodes were calendared and punched into disks with a diameter of 14 mm, and further dried as the target cathode film with a loading of around 5 mg cm^−2^. The CR2032‐type coin cells were assembled with cathode contacting layer‐coated cathode, middle layer SPE, and anode contacting layer‐coated Li metal anode in an argon‐filled glove box. They were manually stacked together and encapsulated. The Li/Li symmetrical cells were prepared by stacking two Li metal anodes with anode contacting layer coatings and middle layer SPE together.

*Sample Characterization and Electrochemical Evaluation*: Thermal stability of the middle layer polymer electrolyte was tested utilizing the TGA from 25 to 500 °C. The crystallinity degree of the middle layer SPE (PEO‐SN‐LiTFSI) and PEO‐LiTFSI were measured by XRD (Rigaku, D/max 2500 X‐ray diffractometer). The surface and cross‐section morphology of the electrolytes and Li anode after cycling were observed by SEM. FTIR spectra of LiCoO_2_ electrolyte were obtained using a Frontier FTIR spectrometer (Perkin‐Elmer) in the transmission mode from 800 to 4000 cm^−1^.

Impedance spectroscopy of the solid polymer electrolytes was tested by EIS using an Autolab PGSTAT 302N system at 60 °C and the spectra were recorded in the frequency range from 10 mHz to 7 MHz with an AC amplitude of 10 mV. Cycling tests were conducted using a LAND CT2001A battery testing system (WuHan, China) at 60 °C.

Ionic conductivity of the solid polymer electrolytes was tested by EIS using an Autolab PGSTAT 302N system at varied temperatures ranging from 30 to 80 °C. The solid polymer electrolyte membranes were sandwiched between two stainless‐steel plate electrodes and the spectra were recorded in the frequency range from 7 MHz to 10 mHz with an AC amplitude of 10 mV. Bulk resistance (*R*b) of membranes was determined from the impedance spectrum. The ionic conductivity was calculated from equation: σ = *L/R*_b_ *S. R*b is the bulk resistance and *L* and *S* are the thickness and area of the solid polymer electrolyte, respectively. The anodic stability (≈4.5 V on the Pt electrode) of the DSM‐SPE was measured by the LSV scan. Cyclic voltammogram (CV) were used to measure the electrochemical stability of electrolytes. XPS (Thermo Scientific ESCA Lab 250Xi) was used to detect the components of SEI and CEI after cycles. All the samples were transferred with the vacuum transfer chambers in case of being exposed to air.

The tLi^+^ of the SPE could be obtained by methods of direct current (DC) polarization combined with EIS, known as the AC (alternating current)/DC method. The values obtained from the method could then be calculated by Equation (1)

(1) $${\mathrm{tLi}}^{+}=I_{\mathrm{ss}}\left(V-I_{0}R_{0}\right)/I_{0}\left(V-I_{\mathrm{ss}}R_{\mathrm{ss}}\right)$$

where *V* is the polarization potential that is applied onto the cell, *I*_0_ and *I*_ss_ are defined as the initial and steady‐state currents, *R*_0_/*R*_ss_ are the initial and steady‐state interfacial resistances before and after the polarization.

## Conflict of Interest

The authors declare no conflict of interest.

## Supporting information

Supplementary
