# Supplementary material for: Differentiated Lithium Salt Design for Multilayered PEO Electrolyte Enables a High‐Voltage Solid‐State Lithium Metal Battery
Source: Adv Sci (Weinh). 2019 Sep 19;6(22):1901036. doi: 10.1002/advs.201901036 (PMC6865005; doi:10.1002/advs.201901036)
Supplement: Supplementary file 1 — Supplementary [file ADVS-6-1901036-s001.pdf]

Copyright WILEY-VCH Verlag GmbH & Co. KGaA, 69469 Weinheim, Germany, 2019.

ADVANCED SCIENCE
Open Access

Supporting Information

for *Adv. Sci.,* DOI: 10.1002/advs.201901036

Differentiated Lithium Salt Design for Multilayered PEO Electrolyte Enables a High-Voltage Solid-State Lithium Metal Battery

Chen Wang, Tao Wang, Longlong Wang, Zhenglin Hu, Zili Cui, Jiedong Li, Shanmu Dong, Xinhong Zhou, and Guanglei Cui**

Supporting Information

Differentiated lithium salt design for multi-layered PEO electrolyte enables high voltage solid state lithium metal battery

Chen Wang, Tao Wang, Longlong Wang, Zhenglin Hu, Zili Cui, Jiedong Li, Shanmu Dong, Xinhong Zhou, Guanglei Cui**

C. Wang, T. Wang, L. Wang, Z. Hu, Z. Cui, J. Li, Prof. S. Dong, Prof. G. Cui

Qingdao Industrial Energy Storage Research Institute, Qingdao Institute of Bioenergy and Bioprocess Technology, Chinese Academy of Sciences, Qingdao 266101, P. R. China

E-mail: dongsm@qibebt.ac.cn, cuigl@qibebt.ac.cn

C. Wang, L. Wang, Z. Hu

Center of Materials Science and Optoelectronics Engineering, University of Chinese Academy of Sciences,

Beijing 100190, P.R. China

Prof. X. Zhou

College of Chemistry and Molecular Engineering, Qingdao University of Science and Technology,

Qingdao 266042, P. R. China

Entry	Cell configuration	Electrolyte	1st-discharge capacity(mAh /g)	Temp. (℃)	Voltage range(V *vs.* Li/Li^+)	Current density	Cycling capacity	Ref.
**1**	Li_3PO_4-mixed LCO/Li	PEO	172	60	3—4.4	0.1C	30th,73%	[1]
**2**	PECA-coated LCO/Li	PEO	172.8	80	2.5—4.45	0.2C	50th,46.3%	[2]
**3**	LCO/Li	P(EO/MEEGE/ AGE)	120	60	3—4.2	0.2C	50th,65.5%	[3]
**4**	LCO/Li	PEO /PMA	119	65	2.5—4.25	0.2C	100th,91.2%	[4]
**5**	5wt%LATP-LCO/Li	PEO	120	60	3—4.2	0.3mA/g	50th,93%	[5]
**6**	LCO/Li	DSM-SPE	150	60	2.5—4.3	0.1C	100th,83.3%	**This work**

Table S1. Summary of cycling performance of high voltage $LiCoO_2$/Li batteries with PEO-based SPE.

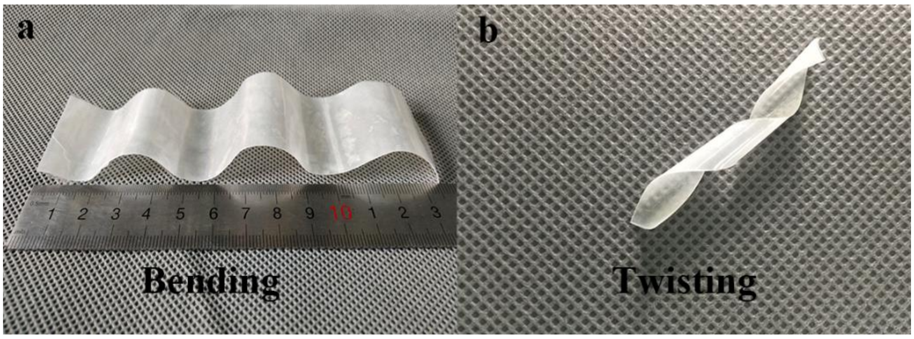

Figure S1. The photographs of the middle layer SPE.

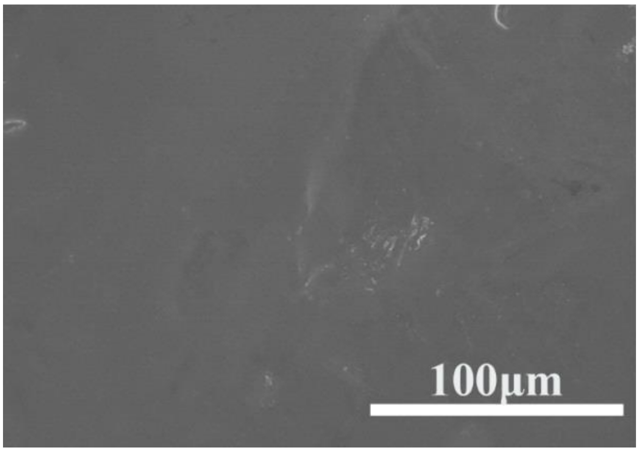

Figure S2. Detailed surface morphology of the middle layer SPE.

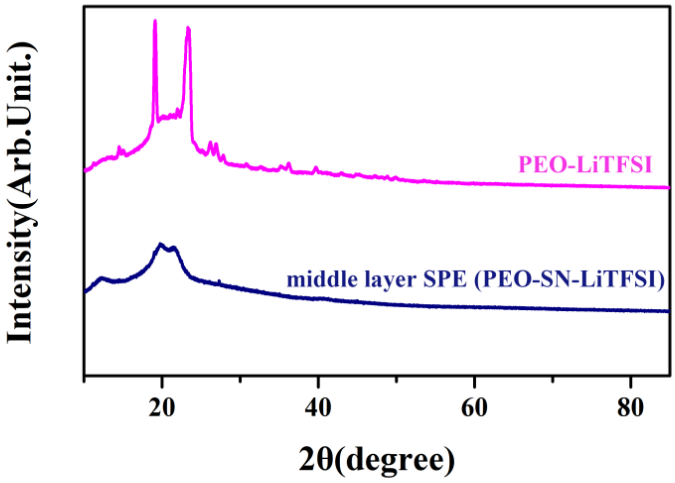

Figure S3. XRD spectra comparison of PEO-LiTFSI and middle layer SPE (PEO-SN-LiTFSI).

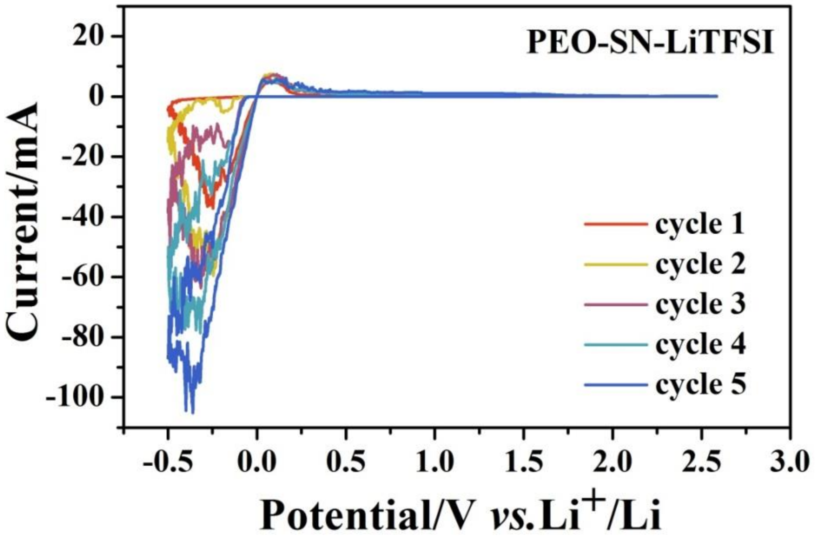

Figure S4. The lithium deposition and stripping stability at lower voltage scanned with PEO-SN-LiTFSI and DSM-SPE from open-circuit voltage to -0.5 V.

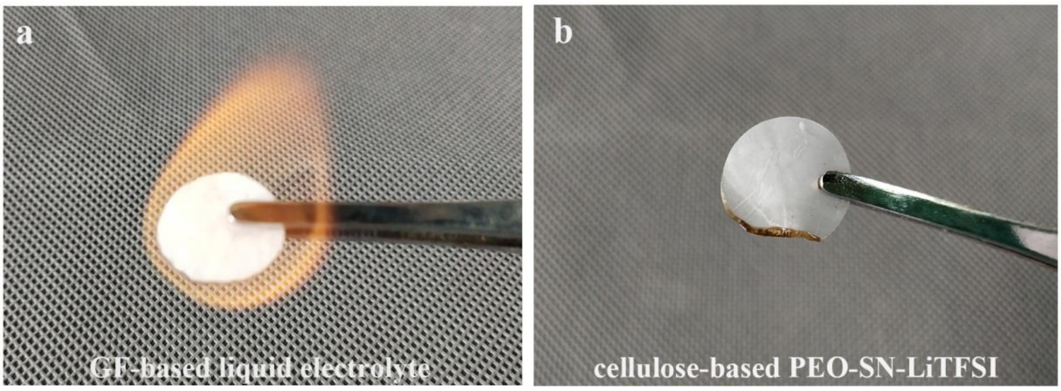

Figure S5. The flame tests of glass fiber-based liquid electrolyte ($LiPF_6$-1M EC/DMC) and cellulose-based PEO-SN-LiTFSI electrolyte.

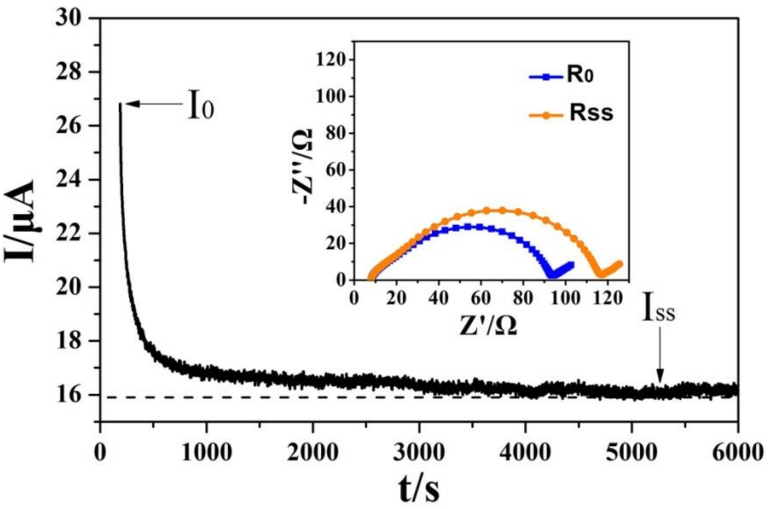

Figure S6. The chronoamperometry profile of a Li/PEO-LiTFSI (5wt% LiTFPFB)/PEO-SN-LiTFSI/PEO-LiTFSI (5wt% LiTFPFB)/Li symmetrical cell under a polarization voltage of 15 mV, and inset is the EISs before and after the polarization.

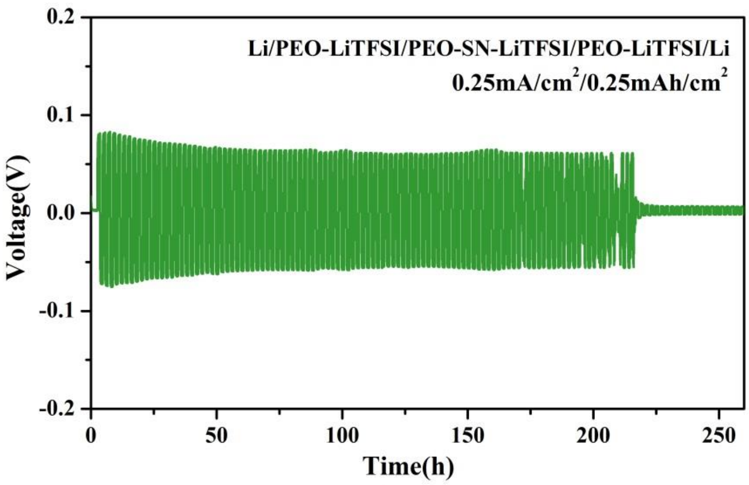

Figure S7. The lithium plating and stripping curve of PEO-LiTFSI/PEO-SN-LiTFSI/PEO-LiTFSI based Li/Li symmetrical cell.

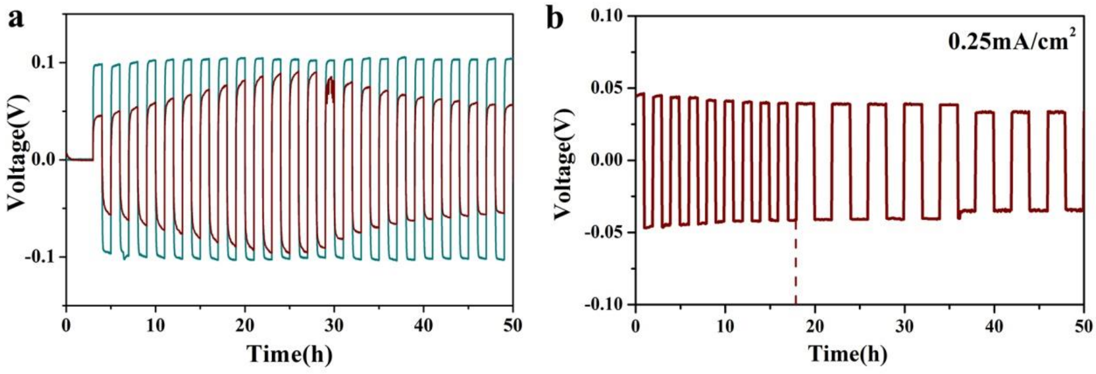

Figure S8. The enlargement of Figure 3(a) and Figure 3(b).

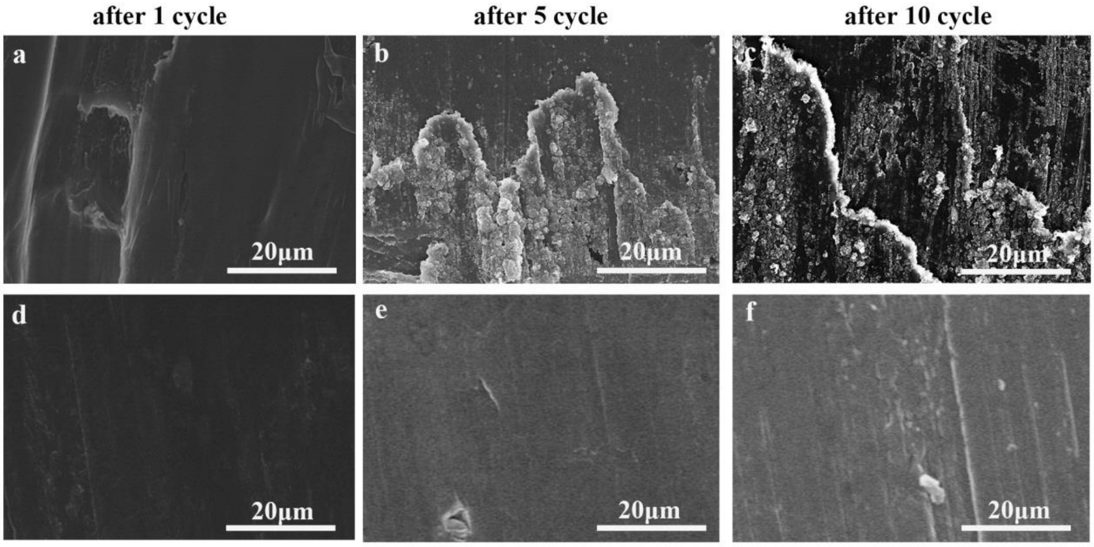

Figure S9. The enlarged SEM images of cross-section for the Li anodes disassembled from the Li/Li symmetrical cells with middle layer SPE (a-c) and with anode contacting layer/middle layer/anode contacting layer SPE (d-f) after 1 cycle, 5 cycle, 10 cycle, respectively.

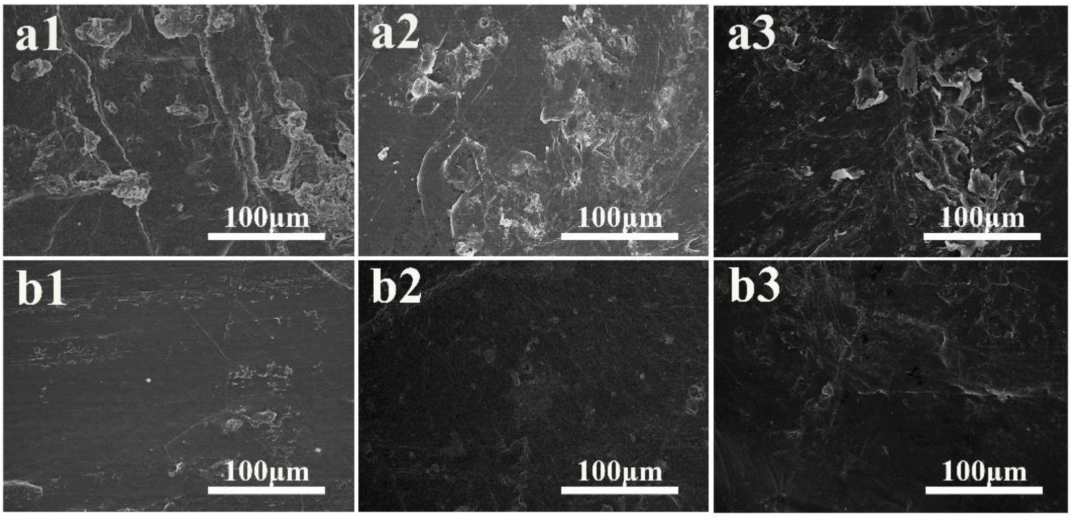

Figure S10. SEM images of the surfaces of Li metal contact to middle layer SPE (a1—a3) and contact to anode contacting layer/middle layer/anode contacting layer SPE (b1—b3) after 1cycle, 5cycle, 10cycle.

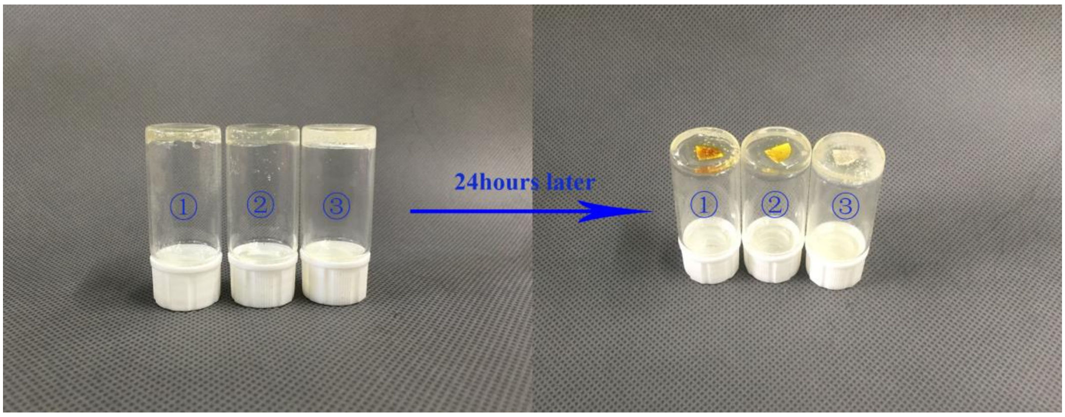

Figure S11. Physical contact reaction between samples of ① PEO-SN, ② PEO-SN-LiTFSI, ③PEO-LiTFSI-5wt%LiTFPFB with Li metal.

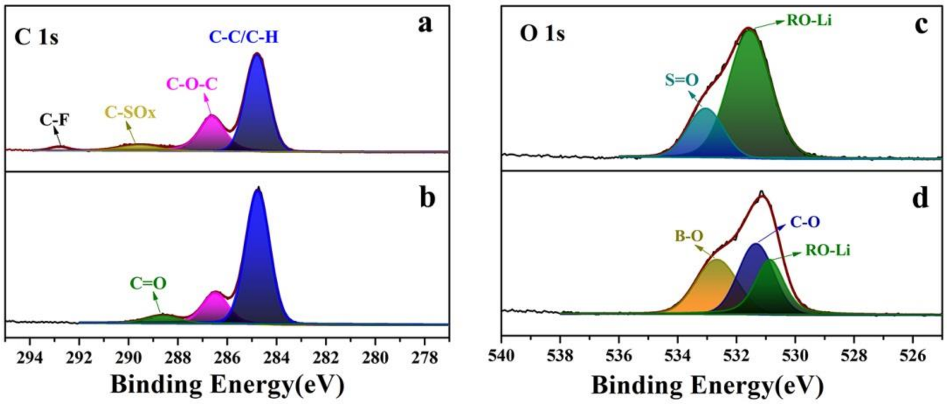

Figure S12. Surface chemistry of the Li metal electrodes contact with (a, c) middle layer SPE and (b, d) DSM-SPE after 10 cycles.

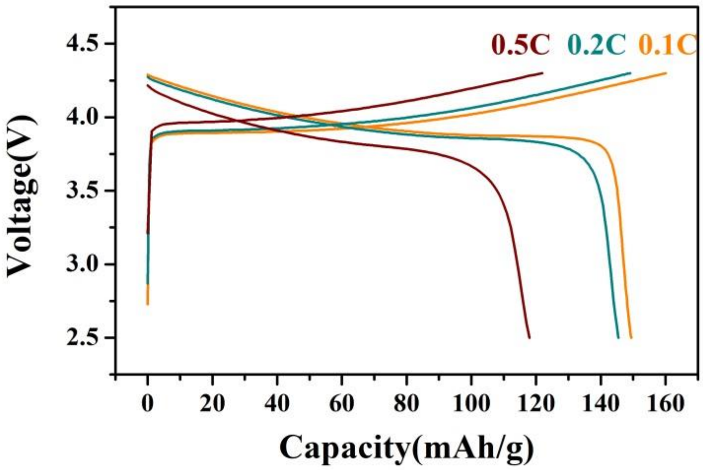

Figure S13. Discharge capacity of $LiCoO_2$/Li battery with DSM-SPE at 0.1C, 0.2C, 0.5C.

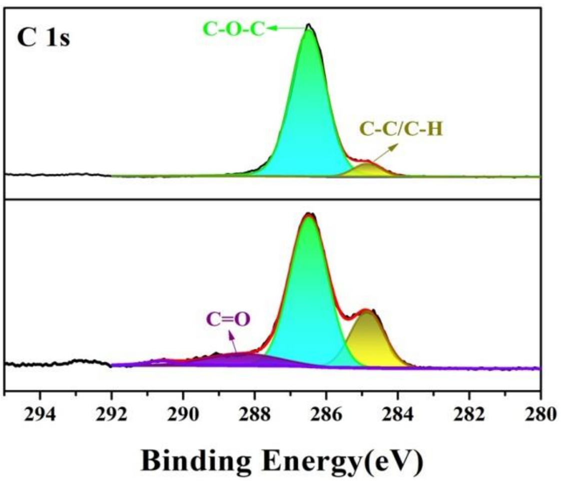

Figure S14. C 1s XPS spectra of cathodes with middle layer SPE and DSM-SPE after 10 cycles.

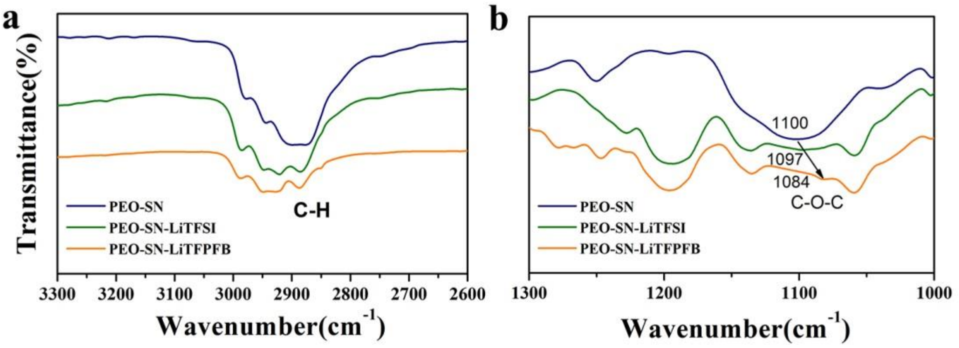

Figure S15. FTIR spectra of PEO-SN, PEO-SN-LiTFSI and PEO-SN-LiTFPFB.

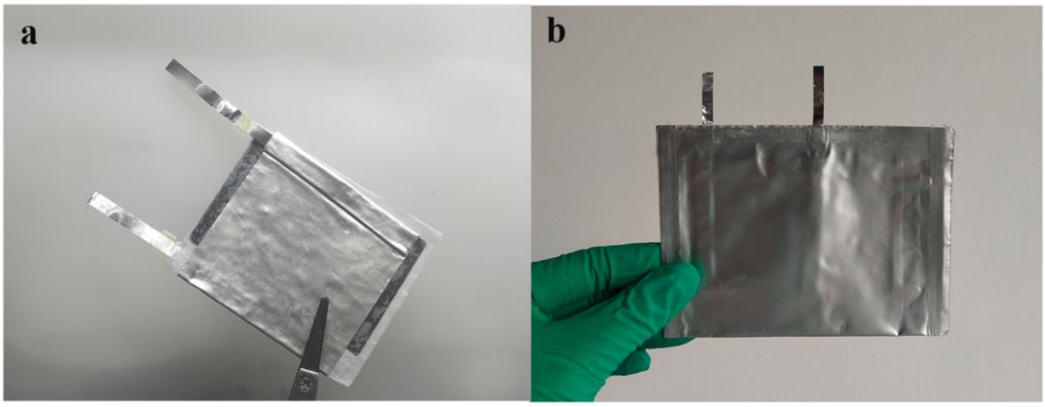

Figure S16. Digital photos of (a) the internal structure and (b) external morphology after packaging of the pouch cell with DSM-SPE.

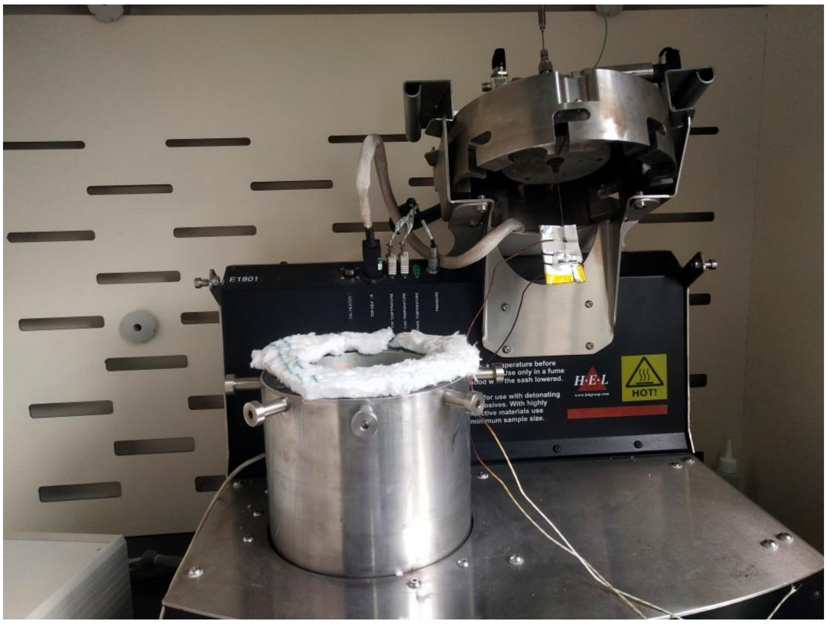

Figure S17. The reactor is the main component of the ARC test stand.

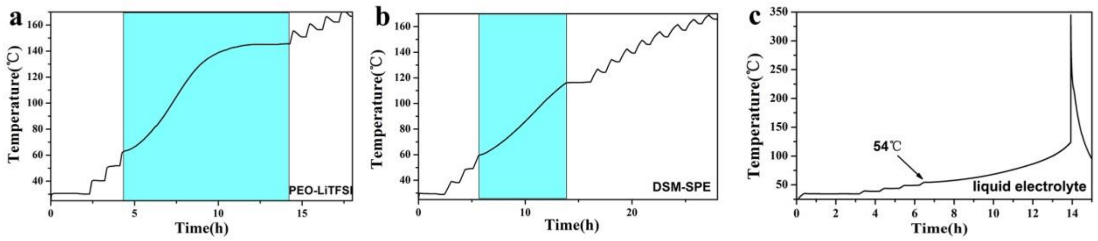

Figure S18. The temperature versus time plots of the LCO/Li metal pouch cells with three kind electrolytes.

[1] S. Seki, Y. Kobayashi, H. Miyashiro, A. Usami, Y. Mita, N. Terada, *Journal of the Electrochemical Society* **2006**, 153, A1073.

[2] J. Ma, Z. Liu, B. Chen, L. Wang, L. Yue, H. Liu, J. Zhang, Z. Liu, G. Cui, *Journal of The Electrochemical Society* **2017**, 164, A3454.

[3] S. Seki, Y. Kobayashi, H. Miyashiro, A. Yamanaka, Y. Mita, T. Iwahori, *Journal of Power Sources* **2005**, 146, 741.

[4] W. Zhou, Z. Wang, Y. Pu, Y. Li, S. Xin, X. Li, J. Chen, J. B. Goodenough, *Adv Mater* **2019**, 31, e1805574.

[5] Q. Yang, J. Huang, Y. Li, Y. Wang, J. Qiu, J. Zhang, H. Yu, X. Yu, H. Li, L. Chen, *Journal of Power Sources* **2018**, 388, 65.
